# *Pachyseris inattesa* sp. n. (Cnidaria, Anthozoa, Scleractinia): a new reef coral species from the Red Sea and its phylogenetic relationships

**DOI:** 10.3897/zookeys.433.8036

**Published:** 2014-08-13

**Authors:** Tullia I. Terraneo, Michael L. Berumen, Roberto Arrigoni, Zarinah Waheed, Jessica Bouwmeester, Annalisa Caragnano, Fabrizio Stefani, Francesca Benzoni

**Affiliations:** 1 Dept. of Biotechnology and Biosciences, University of Milano-Bicocca, Piazza della Scienza 2, 20126 Milan, Italy; 2 Red Sea Research Center, King Abdullah University of Science and Technology (KAUST), Thuwal, Saudi Arabia; 3 Department of Marine Zoology, Naturalis Biodiversity Center, P.O. Box 9517, 2300 RA Leiden, The Netherlands; 4 Borneo Marine Research Institute, Universiti Malaysia Sabah, Jalan UMS, 88400 Kota Kinabalu, Sabah, Malaysia; 5 Water Research Institute–National Research Council (IRSA-CNR), Via del Mulino 19, I-20861 Brugherio, Italy; 6 Institut de Recherche pour le Développement, UMR227 Coreus2, 101 Promenade Roger Laroque, BP A5, 98848 Noumea Cedex, New Caledonia

**Keywords:** *Pachyseris rugosa*, *Pachyseris speciosa*, *Leptoseris foliosa*, micro-morphology, COI-16S-rRNA intergenic spacer, taxonomy

## Abstract

A new scleractinian coral species, *Pachyseris inattesa* **sp. n.**, is described from the Red Sea. Despite a superficial resemblance with some species in the agariciid genus *Leptoseris* with which it has been previously confused, *P. inattesa* **sp. n.** has micro-morphological characters typical of the genus *Pachyseris*. This genus, once part of the Agariciidae, is comprised of five extant species and is widely distributed throughout the tropical Indo-Pacific. It is currently *incertae sedis* as a result of recent molecular analysis and appears to be closely related to the Euphylliidae. A molecular phylogenetic reconstruction including *P. inattesa* **sp. n.**, the genus type species *P. rugosa*, and *P. speciosa*, all present in the Red Sea, was performed using the mitochondrial intergenic spacer between COI and 16S-rRNA. The results confirm that *P. inattesa* **sp. n.** is a monophyletic lineage closely related to the other *Pachyseris* species examined.

## Introduction

The zooxanthellate and reef-dwelling hard coral genus *Pachyseris* Milne-Edwards & Haime, 1849 is widely distributed throughout the Indo-Pacific, from the Red Sea to the Marshall Islands, Samoa, and Tahiti (Veron and Pichon 1980, Scheer and Pillai 1983, Sheppard and Sheppard 1991, Veron 2000). It has previously been ascribed to the family Agariciidae Gray, 1847 (Vaughan and Wells 1943, Wells 1956, Veron and Pichon 1980) on the basis of macro-morphological affinities with genera like *Gardineroseris* Scheer & Pillai, 1974 and *Pavona* Lamarck, 1801 (Veron and Pichon 1980). However, based on recent molecular analyses of combined mitochondrial cytochrome oxidase I (COI) and cytochrome b DNA sequences, it was discovered that the genus *Pachyseris* is not even closely related to the agariciid genera *Agaricia* Lamarck, 1801, *Gardineroseris*, *Leptoseris* Milne-Edwards & Haime, 1849, and *Pavona* and that it is basal to the family Euphylliidae Alloiteau, 1952 (Fukami et al. 2008, Kitahara et al. 2010). To date, the phylogenetic position of *Pachyseris* remains unclear and this genus is currently regarded as *incertae sedis*.

Throughout the last two centuries, more than ten nominal species of *Pachyseris* have been described, probably overestimating the true number of the actual species due to the high intraspecific variability of the species (Matthai 1948, Nemenzo 1955, Veron and Pichon 1980). This genus currently includes five extant species (Veron 2000), namely *Pachyseris rugosa* (Lamarck, 1801), the type species, *Pachyseris speciosa* (Dana, 1846), *Pachyseris involuta* (Studer, 1878), *Pachyseris gemmae* Nemenzo, 1955, and *Pachyseris foliosa* Veron, 1990. In the Red Sea, two species of the genus *Pachyseris*, *Pachyseris speciosa* and *Pachyseris rugosa*, have been recorded (Scheer and Pillai 1983, Sheppard and Sheppard 1991).

Although the Red Sea is known to be an important region of biodiversity and endemism (Ekman 1953, Briggs 1974, Stehli and Wells 1971, Ormond and Edwards 1987), it has remained largely understudied. Despite the fact that the region has an early history of scientific works and attracted particular interest among taxonomists (e.g. Forskål 1775, Ehrenberg 1834, Klunzinger 1879a, 1879b, Scheer and Pillai 1983, Sheppard and Sheppard 1991), there is a lack of available recent works on the diversity and systematics of Red Sea fauna outside of the Gulf of Aqaba (Berumen et al. 2013). Within this context, the project entitled “Biodiversity in the Saudi Arabian Red Sea” was initiated by the King Abdullah University of Science and Technology (KAUST) in 2012. Two initial cruises were held in March and September 2013 to sample scleractinian corals at different localities along the coast of the Saudi Arabian Red Sea for the purpose of establishing a reference collection. Several colonies of a coral previously identified as *Leptoseris tenuis* Van der Horst, 1921 by Scheer and Pillai (1983) and *Leptoseris foliosa* Dinesen, 1980 by Sheppard and Sheppard (1991) were collected. Interestingly, the study of the original descriptions and type material illustrations of these species revealed that the collected material is different from any species in the genus *Leptoseris*, and that it has some typical features of *Pachyseris* despite superficially appearing different from the nominal species in this genus as described in the taxonomic literature (Matthai 1948, Nemenzo 1955, Veron and Pichon 1980, Veron 2000). As a result, this species is hereby described as *Pachyseris inattesa* sp. n. In order to evaluate the phylogenetic relationships of this new species with respect to other species of *Pachyseris* and *Leptoseris*, we sequenced the mitochondrial non-coding spacer between COI and 16S-rRNA (Luck et al. 2013) of *Pachyseris inattesa* sp. n., *Pachyseris speciosa*, *Pachyseris rugosa*, and *Leptoseris foliosa*, and compared them with the agariciids *Leptoseris incrustans* (Quelch, 1886), *Leptoseris tubulifera* Vaughan, 1907, *Leptoseris hawaiiensis* Vaughan, 1907, *Leptoseris papyracea* (Dana, 1846), *Leptoseris mycetoseroides* Wells, 1954, *Leptoseris scabra* Vaughan, 1907, *Pavona clavus* (Dana, 1846), and *Pavona varians* Verrill, 1864.

## Methods

### Sampling and specimen identification

Several colonies of *Pachyseris* were collected at various localities in the Red Sea and in the Indo-Pacific. Digital images of living corals *in situ* were taken with a Canon G9 in an Ikelite underwater housing or a Nikon Coolpix 7900 in a Nikon WP-CP4 waterproof case. From each coral specimen collected, a 2 cm^2^ fragment was preserved in either 95% ethanol or CHAOS solution (Sargent et al. 1986, Fukami et al. 2004) for molecular analyses. After tagging, the remaining corallum was bleached in sodium hypochlorite for 48h to remove soft parts, rinsed in freshwater, and air-dried for morphological analyses. Images of the cleaned skeletons were taken with a Canon G9 digital camera. Samples were identified following Veron and Pichon (1980), Scheer and Pillai (1983), Veron (1986), Sheppard and Sheppard (1991), and Nishihira and Veron (1995). Two colonies of *Leptoseris foliosa* from New Caledonia already studied by Benzoni et al. (2012) were also included in the following analyses.

### Morphological analyses

Macro and micro-morphological characters of *Pachyseris* samples were examined using both light microscopy (Zeiss Stemi DV4 stereo-microscope) and scanning electron microscopy (SEM). For SEM, *Pachyseris speciosa*, *Pachyseris rugosa*, and *Leptoseris foliosa* fragments were ground, mounted on stubs using silver glue, sputter-coated with conductive gold film, and examined using Vega Tescan Scanning Electron Microscopy at the SEM Laboratory, University of Milano-Bicocca. Fragments of *Pachyseris inattesa* sp. n. (specimens KAUST SA492 and KAUST SA1305) were sputter-coated with Au-Pd and imaged using a Quanta 200 FEG SEM at the King Abdullah University of Science and Technology.

### DNA extraction, PCR amplification, and sequence analyses

Twenty *Pachyseris* and two *Leptoseris foliosa* specimens were included in the molecular analyses (Table 1). Total DNA was extracted using DNAeasy® Tissue kit (Qiagen Inc., Valencia, CA, USA) for samples stored in ethanol according to the manufacturer’s protocol and a phenol-chloroform based method for samples in CHAOS (Fukami et al. 2004). Each extracted sample was quantified using a NanoDrop® 1000 spectrophotometer (Thermo Scientific) and diluted to a final concentration of 3 ng/ml. The mitochondrial intergenic spacer between COI and 16S-rRNA (hereafter IGR), previously used to evaluate species boundaries within the family Agariciidae (Luck et al. 2013), was chosen as a marker and amplified using newly designed primers AGAH (5’- GCT TGA CAG GGT TTC CAA GA - 3’) and AGAL (5’- CGC ATT GAA ACA CGA GCT TA - 3’). The same region was amplified independently by Luck et al. (2013) in agariciids, but when starting our analyses, primers designed by them for this mitochondrial intergenic spacer were still not available in literature. Reactions were conducted in a 25 µl PCR mix, composed of 1X PCR buffer, 2 mM MgCl_2_, 0.4 µM of each primer, 0.1 mM dNTP mix, 2 U taq polymerase (Sigma-Aldrich Co., St. Louis, MO, USA), and 4 µl of DNA solution (10-30 ng of DNA). The thermal cycle consisted of a first denaturation phase at 94°C for 4 min, followed by 30 cycles to 94 °C for 1 min, 54 °C for 1 min, 72 °C for 1 min, and finally an elongation phase of 72 °C for 5 min. All samples were purified with Illustra ExoStar (GE Healthcare, Buckinghamshire) and directly sequenced in forward and reverse directions using an ABI 3130xl Genetic Analyzer (Applied Biosystems). Sequences produced in this study have been deposited at EMBL and accession numbers are listed in Table 1.

**Table 1.** List of samples used in this study. For each specimen the registration code, identification, collection locality, collector, and EMBL accession number are provided.

Code	Identification	Locality	Collector	IGR between COI and 16S-rRNA
BMRI 62	*Pachyseris rugosa*	Sabah, Malaysia	Waheed Z.	LK934487
HS2856	*Pachyseris rugosa*	New Caledonia	Benzoni F.	LK934488
HS2594	*Pachyseris rugosa*	New Caledonia	Benzoni F.	LK934489
SO 040	*Pachyseris speciosa*	Socotra Island, Yemen	Benzoni F.	LK934490
SO 020	*Pachyseris speciosa*	Socotra Island, Yemen	Benzoni F.	LK934491
MA 439	*Pachyseris speciosa*	Maldives	Benzoni F.	LK934492
MA 476	*Pachyseris speciosa*	Maldives	Benzoni F.	LK934493
MA 477	*Pachyseris speciosa*	Maldives	Benzoni F.	LK934494
SA 376	*Pachyseris speciosa*	Red Sea, Saudi Arabia	Benzoni F.	LK934495
BMRI 06	*Pachyseris speciosa*	Sabah, Malaysia	Waheed Z.	LK934496
BMRI 66	*Pachyseris speciosa*	Sabah, Malaysia	Waheed Z.	LK934497
BMRI 68	*Pachyseris speciosa*	Sabah, Malaysia	Waheed Z.	LK934498
RMNH Coel. 41613	*Pachyseris inattesa* sp. n.	Red Sea, Saudi Arabia	Benzoni F.	LK934499
SA 004	*Pachyseris inattesa* sp. n.	Red Sea, Saudi Arabia	Benzoni F.	LK934500
SA 1284	*Pachyseris inattesa* sp. n.	Red Sea, Saudi Arabia	Benzoni F.	LK934501
SA 1301	*Pachyseris inattesa* sp. n.	Red Sea, Saudi Arabia	Benzoni F.	LK934502
SA 1305	*Pachyseris inattesa* sp. n.	Red Sea, Saudi Arabia	Benzoni F.	LK934503
SA 887	*Pachyseris inattesa* sp. n.	Red Sea, Saudi Arabia	Benzoni F.	LK934504
SA 1300	*Pachyseris inattesa* sp. n.	Red Sea, Saudi Arabia	Benzoni F.	LK934505
SA 1293	*Pachyseris inattesa* sp. n.	Red Sea, Saudi Arabia	Benzoni F.	LK934506
HS2854	*Leptoseris foliosa*	New Caledonia	Benzoni F.	LK934507
HS2873	*Leptoseris foliosa*	New Caledonia	Benzoni F.	LK934508

Phylogenetic relationships between species were inferred using our sequences and 15 sequences of Agariciidae downloaded from GenBank based on Luck et al. (2013) in order to define the position of *Pachyseris inattesa* sp. n. with respect to the genus *Leptoseris*. Moreover, in order to root the mtDNA phylogeny, we selected *Siderastrea radians* (Pallas, 1776) (clade IX *sensu* Fukami et al. 2008) as outgroup based on the phylogeny proposed by Fukami et al. (2008). Sequences were viewed, edited, and assembled using CodonCode Aligner 4.2.5 (CodonCode Corporation, Dedham, MA, USA) and manually checked using BioEdit 7.2.5 (Hall 1999). Multiple alignments were carried out using the E-INS-i option in MAFFT 7.110 (Katoh et al. 2002, Katoh and Standley 2013) under default parameters. Invariable, polymorphic, and parsimony informative sites were detected with DnaSP 5.10.01 (Librado and Rozas 2009). Intra- and inter-specific pairwise distances (uncorrected *p*-distances) were calculated in MEGA 4.0.2 (Tamura et al. 2007). Phylogenetic relationships were reconstructed using Bayesian Inference (BI), Maximum Likelihood analyses (ML), and Maximum Parsimony (MP). Bayesian Inference was conducted with MrBayes 3.1.2 (Huelsenbeck and Ronquist 2001, Ronquist and Huelsenbeck 2003), Maximum Likelihood analyses using PhyML 3.0 (Guindon and Gascuel 2003), and Maximum Parsimony was based on PAUP* 4.0b10 (Swofford 2003). The best-fit substitution model was determined using the Akaike Information Criterion (AIC) as implemented in MrModeltest 2.3 (Posada and Crandall 1998) in conjunction with PAUP 4.0b10 (Swofford 2003). AIC identified the General Time Reversible (GTR) model with gamma distributed rate variation among sites (+Γ) (Γ = 1.02) as the most suitable model. Bayesian Inference analyses consisted of four parallel Markov Chains Monte Carlo (MCMC) implemented for 1,000,000 generations, saving a tree every 100 generations and discarding the first 2,501 trees as burn-in. Bayesian Inference analysis was stopped when the standard deviations of split frequencies were <0.01. As an additional tool, the software Tracer 1.5 (Drummond and Rambaut 2007) was used to verify the convergence of parameters and correctly estimate the burn-in. Finally, clade support was assessed based on posterior probability. The Best Maximum Likelihood tree was reconstructed with PhyML using the default parameters and 1,000 bootstrap replicates to verify the robustness of the internal branches of the tree. Maximum Parsimony was conducted using the TBR branch swapping method with 1000 replications, random addition for 10 replicates, nchuck = 100, chuckscore = 1. Node supports were obtained with 1,000 bootstrap replicates.

### Abbreviations

BIBELOT IRD Biodiversité Benthique dans les iles Loyauté Expedition, Loyalty Islands, New Caledonia, 2014

BMRI Borneo Marine Research Institute, Universiti Malaysia Sabah, Malaysia

IRD Institut de Recherche pour le Développement, Nouméa, New Caledonia

KAUST King Abdullah University of Science and Technology, Thuwal, Saudi Arabia

KBEA KAUST Biodiversity Expedition to the Gulf of Aqaba, 2013

KBEF KAUST Biodiversity Expedition to the Farasan Banks and Farasan Islands, 2013

NIUGINI Niugini Biodiversity Expedition, Papua New Guinea, 2012

RMNH Coel. Rijksmuseum van Natuurlijke Historie, Coelenterate collection, Naturalis Biodiversity Center, Leiden, The Netherlands

SMEE Semporna Marine Ecological Expedition, 2010

TOE Tara Oceans Expedition, 2009-2012

UNIMIB University of Milano-Bicocca, Milan, Italy

USNM United States National Museum of Natural History, Washington DC, USA

## Systematic section

### Order Scleractinia Bourne, 1905
*Incertae sedis*

#### Genus Pachyseris

Taxon classificationAnimaliaScleractiniaAgariciidae

Milne-Edwards & Haime, 1849

##### Type species

(by monotypy). *Agaricia rugosa* Lamarck, 1801.

#### Pachyseris rugosa

Taxon classificationAnimaliaScleractiniaAgariciidae

(Lamarck, 1801)

Figures 1a–c , 2

Pachyseris rugosa For synonymy, see Scheer and Pillai (1983).

##### Material examined.

BMRI 62, Semporna, Malaysia (MV Celebes, Explorer, SMEE), 04°34'01.8"N, 118°45'27.5"E, 11 December 2010, coll. Z. Waheed; IRD HS2856, Prony Bay, New Caledonia, 22°21.230'S, 166°49.300'E, 10 m, 23 February 2011, coll. F. Benzoni; IRD HS2893, Prony Bay, New Caledonia, 22°21.230'S, 166°49.300'E, 10 m, 22 February 2011, coll. F. Benzoni; IRD HS3383, Maré, Loyalty Islands, New Caledonia (MV Alis, BIBELOT), 16 February 2014, coll. F. Benzoni.

**Figure 1.**
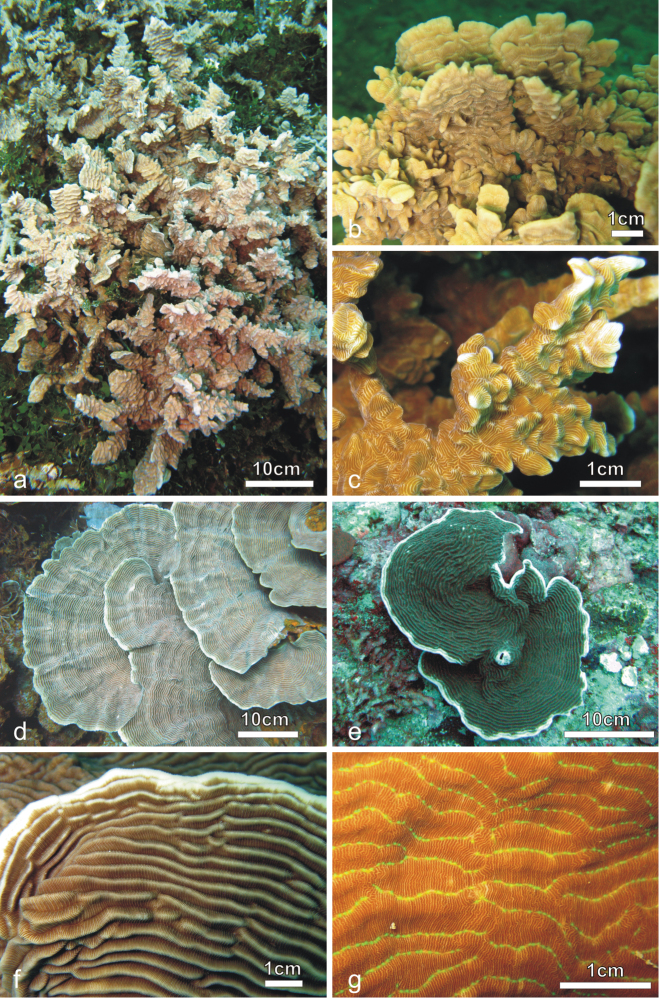
Colonies of *Pachyseris rugosa* (**a–c**) and *Pachyseris speciosa* (**d–g**) *in situ*. **a** Image of the whole colony of specimen IRD HS2893, Prony Bay, New Caledonia **b** Lateral view of the fronds of specimen IRD HS2594, Prony Bay, New Caledonia **c** Fronds of specimen IRD HS2856 viewed from above, Prony Bay, New Caledonia **d** Tiers of foliose projections of a colony from New Caledonia **e** Image of specimen UNIMIB SO040, Socotra Island **f** Part of specimen KAUST SA714, Saudi Arabia **g** Detail of a colony with reduced carinae and brightly colored polyp mouths, growing in very turbid environment, Banc des Japonais, New Caledonia.

**Figure 2.**
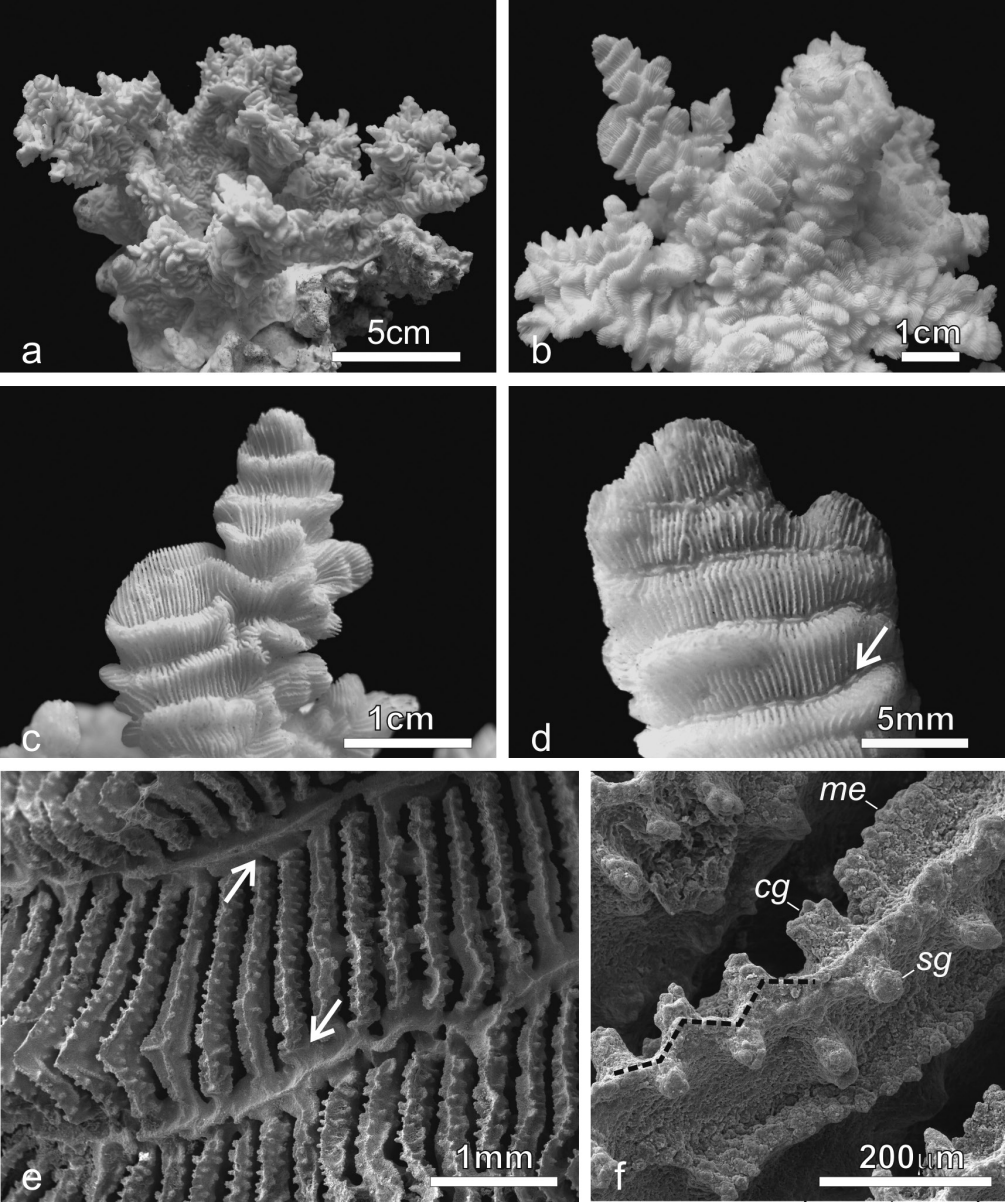
*Pachyseris rugosa*. **a** Lateral view of colony IRD HS1442 **b** Lateral view of colony IRD HS152 showing very irregular fronds and carinae **c** Detail of the fronds of specimen IRD HS152 **d** Specimen IRD HS221, white arrow points at a dash-like columella **e** SEM image of IRD HS2594, white arrows point at the fused dissepiments connecting the inner end of the radial elements and the reduced columellae **f** SEM image of ornamentation on radial elements showing single granules (**sg**), clumped granules (**cg**) and menianae (**me**).

*Corallum*: Highly variable in shape from encrusting with foliose margins and central knobs (Veron and Pichon 1980) to caespitose with bifacial fronds of variable width growing upward (Figures 1a–c, 2a–b). Fronds can be anastomose (Veron and Pichon 1980). The corallum surface is undulated or corrugated due to the presence of well-developed carinae (Veron and Pichon 1980).

*Calices*: Arranged in rows, mostly indistinct. Rows can be long and continuous, or short and irregular, especially on the fronds (Figures 2a–d). Series of calices are generally arranged parallel to each other and are concentric in the encrusting or foliose parts of the corallum. Series are separated by carinae with variable vertical development and inclination with respect to the corallum surface (Figures 2b–c). At the base of the fronds, the carinae can be very short and resemble hydnophoroid protuberances (Figure 1c).

*Columella*: Well-developed, made by a dash-like process rising from a horizontal fig made of dissepiments from the inner ends of the radial elements (Figure 2d; Veron and Pichon 1980: fig. 137). In the terminal parts of the corallum, especially on the top of the fronds, dash-like processes can be reduced to low-lying ridges mostly continuous between centres and the structure made by the fused dissepiments is more clearly visible (Figure 2e).

*Radial elements*: Radial elements are continuous across the carinae, regularly spaced and equal or slightly alternating (Figures 2c–e). Lateral faces bear regularly distributed, parallel lines of granules or/and ledge-like features called menianae (Benzoni et al. 2012, Kitahara et al. 2012a) often alternating along the same radial element. Such lateral ornamentation is variable and includes single granules, groups of 2-4 clumped granules and menianae with minutely beaded edges (Figure 2f). All lateral ornamentations are oriented in lines parallel to the upper radial element margin. The upper margin of the radial element is minutely beaded and straight in the portions above the menianae and typically attains a zigzag pattern with variable angles in the portions where single or clumped granules are present (Figure 2f).

#### Pachyseris speciosa

Taxon classificationAnimaliaScleractiniaAgariciidae

(Dana, 1846)

**Figure 3.**
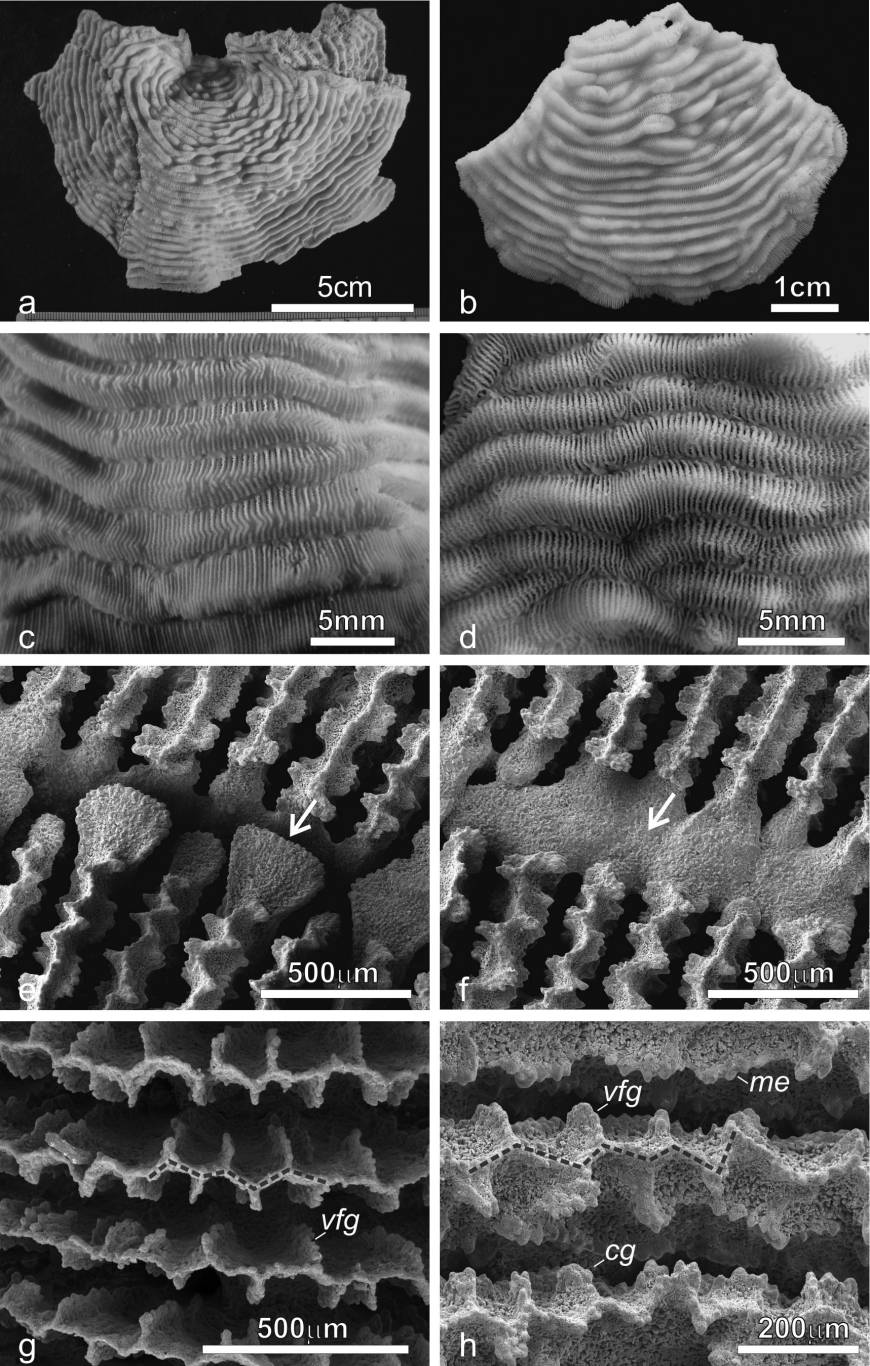
*Pachyseris speciosa*. **a** The holotype USNM 119, West Indies **b** Specimen UNIMIB SO020, Socotra Island **c** Detail of the holotype showing parallel carinae and alternating radial elements **d** Detail of the specimen in **b e** SEM image of IRD HS2673, white arrows point at one of the spatula-shaped processes extending from the inner end of the radial elements and forming the columella **f** SEM image of the same specimen (Figure 3e) in a portion where columella processes are fused to form a continuous structure (white arrow) **g** SEM image of IRD HS2263 showing the ornamentation of the radial elements consisting of vertically fused granules (vfg) seen from above and the zigzag pattern of the radial element margin (dashed line) **h** SEM image of IRD HS2673 showing the radial element zigzag pattern (dashed line) and face ornamentation consisting of clumped granules (**cg**), vertically fused granules (**vfg**), and menianae (**me**).

Figures 1d–g , 3 , 11a, d

Pachyseris speciosa For synonymy, see Scheer and Pillai (1983).

##### Material examined.

**Holotype:** USNM 119 (Figures 2a, c). Type Locality: East Indies (U.S. Exploring Expedition).

##### Other material.

KAUST SA376, Shi'b Nazar, Saudi Arabia, 22°19'60.00"N, 38°51'15.78"E, 16 March 2013, coll. F. Benzoni; KAUST SA714, Ras Al-Ubayd, Saudi Arabia (MV Dream-Master, KBEA), 26°44.167'N, 36°02.659'E, 26 September 2013, coll. F. Benzoni; UNIMIB TO-DJ240, Obock, Djibouti (MV Tara, TOE), 11°57.517'N, 43°18.787'E, 3 February 2010, coll. F. Benzoni; UNIMIB TO-DJ341, Arta region, Djibouti (MV Tara, TOE), 11°35.365'N, 42°52.560'E, 10 February 2010, coll. F. Benzoni; UNIMIB SO020, Roosh, Socotra Island, 12°37.237'N, 54°21.090'E, 12 March 2010, coll. F. Benzoni; UNIMIB SO040, Ras Adho, Socotra Island, 12°38.638'N, 54°16.147'E, 13 March 2010, coll. F. Benzoni; BMRI 66, Semporna, Malaysia (MV Celebes Explorer, SMEE), 04°36'10.0"N, 118°45'53.6"E, 11 December 2010, coll. Z. Waheed; BMRI 68, Semporna, Malaysia, (MV Celebes Explorer, SMEE), 04°37'32.2"N, 118°40'58.0"E, 12 December 2010, coll. Z. Waheed; UNIMIB PFB342, Madang, Papua New Guinea (MV Alis, NIUGINI), 05°05.854'S, 145°48.525'E, 20 November 2012, coll. F. Benzoni; IRD HS2594, Ilot N'do, Nouméa, New Caledonia, 15 May 2009, coll. F. Benzoni.

*Corallum*: Unifacial and encrusting with foliose margins (Figure 1e) to laminar, sometimes forming tiers of laminae (Figure 1d). The corallum surface is corrugated due to the presence of mostly concentric continuous carinae (Figures 1d–g, 3a–d).

*Calices*: Arranged in rows, mostly indistinct although sometimes polyp mouths can have a distinct coloration *in vivo* (Figure 1g) allowing recognition of the position of the single calice underneath. Rows generally long and continuous (Figures 1d–g, 3a–d). Series of calices are arranged parallel to each other, concentric and separated by carinae with variable vertical development and inclination with respect to the corallum surface (Figures 1d–g, 3a–d). When asymmetrical, carinae are inclined towards the margin of the corallum.

*Columella*: Well-developed, low-lying in the valleys between carinae formed by the fusion of spatula-shaped processes extending from the inner end of the radial elements (Figure 3e). Radial elements of the higher order form larger processes alternating with the smaller ones from the elements of lower order (Figure 3e). In the same series of calices the processes forming the columella can be separate (Figure 3e) or completely fused (Figure 3f).

*Radial elements*: Radial elements are continuous across the carinae, regularly spaced and equal or slightly alternating (Figure 3c–d). Lateral faces bear regularly distributed, parallel lines of granules or/and menianae. Such lateral ornamentation is variable and includes groups of clumped granules (Figure 2h), menianae with minutely beaded edges and vertically fused granules forming structures similar to menianae but oriented perpendicularly rather than parallel to the radial element margin (Figure 3g–h). The upper margin of the radial elements is minutely beaded and typically attains a zigzag pattern with lateral ornamentations at the angles (Figure 3g–h). This pattern can be so pronounced in some specimens as to give the radial elements a “wavy or even crenellated” appearance to the naked eye (Veron and Pichon 1980: 84; Scheer and Pillai 1983).

#### Pachyseris inattesa

Taxon classificationAnimaliaScleractiniaAgariciidae

Benzoni & Terraneo sp. n.

http://zoobank.org/4C6008D7-FF14-47CA-B65D-7E65F88C477D

**Figure 4.**
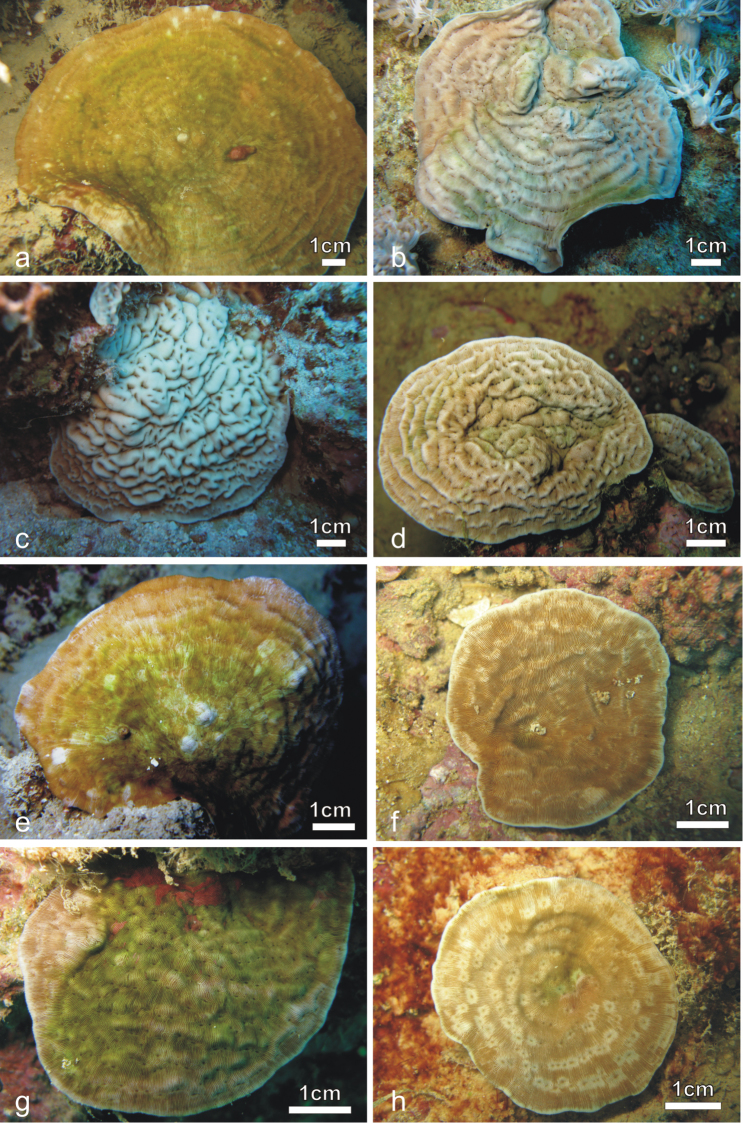
*Pachyseris inattesa* sp. n. *in situ*. **a** KAUST SA1300 **b** KAUST SA426 **c** KAUST SA887 **d** holotype RMNH Coel. 41613 **e** KAUST SA1284 **f** KAUST SA004 **g** KAUST SA1301 **h** KAUST SA1305.

Figures 4 [Fig F5] [Fig F6] –7 , 10a, c, e , 11b–e

Leptoseris tenuis Van der Horst, 1921 (partim). Scheer and Pillai 1983: figs 7–11.
Leptoseris foliosa Dinesen, 1980. Sheppard and Sheppard 1991: fig. 85, Plate 58; Veron 2000: fig. 8.

##### Material examined.

**Holotype:** RMNH Coel. 41613 (Figures 4d, 6b–d). Type Locality: Al Lith, Saudi Arabia (MV Dream-Master, KAUST Biodiversity Cruise to the Farasan Banks and Farasan Islands), 20°07.690'N, 40°12.513'E, 3 March 2013, coll. F. Benzoni.

**Figure 5. F5:**
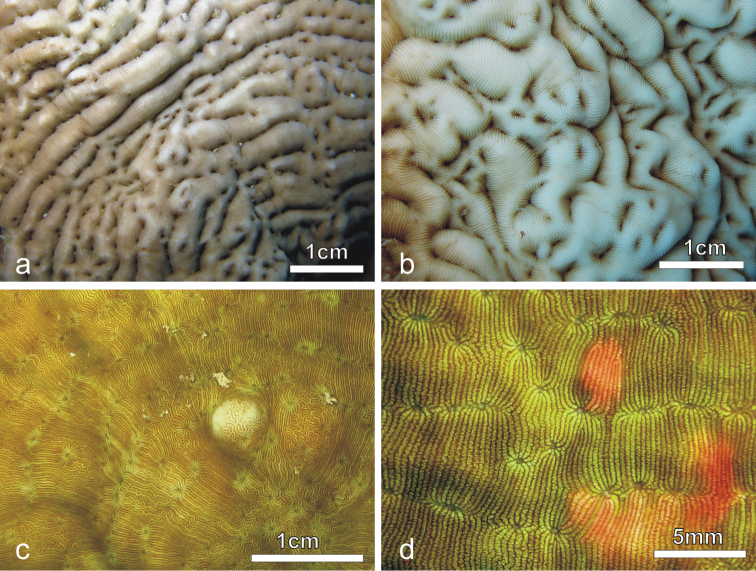
Development of carinae and series of calices in *Pachyseris inattesa* sp. n. *in situ*. **a** Well-developed carinae and elongated series of calices in specimen KAUST SA429 **b** Well-developed carinae and short series of calices in specimen KAUST SA887 **c** Poorly developed carinae and short series of calices in specimen KAUST SA1284 **d** Poorly developed carinae and long series of calices in specimen KAUST SA1293.

**Figure 6. F6:**
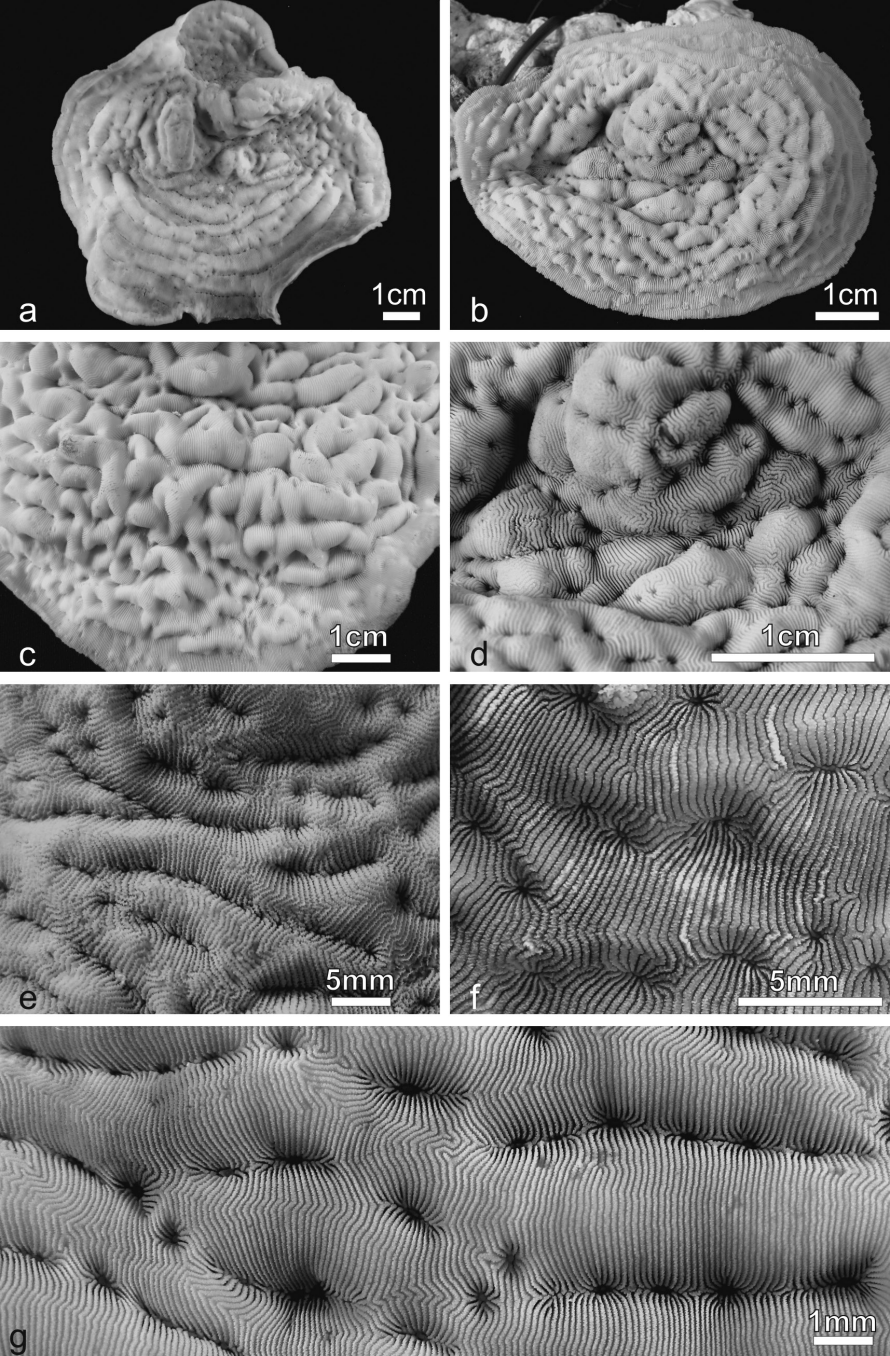
*Pachyseris inattesa* sp. n. **a** KAUST SA426 **b** Holotype RMNH Coel. 41613 **c** View of the marginal part of the holotype in **b d** View of the central part of the holotype in **b e** KAUST SA678 **f** KAUST SA1301 **g** KAUST SA429.

*Corallum*: The specimen is 1.5 cm high from the base in its original growth position, and 7.5 × 5.2 cm wide. The holotype is oval-shaped, attached at the centre with free margins and sunken in the central part (Figures 4d, 6b). At the opposite ends of its largest diameter the corallum margins bend in different directions with respect to the plane of the central encrusting part (upwards at the left-hand side and downwards at the right-hand side of Figure 6b). The corallum surface is irregularly undulated due to the presence of well-developed carinae, which are symmetrical and thick in the central part (Figure 6d) and become increasingly shorter, lower, and more inclined towards the margins (Figure 6c).

*Calices*: Arranged in short rows, mostly distinct (Figure 6d) especially towards the margins where the series become shorter (Figure 6c). Calices and series of calices are arranged parallel to each other, concentric and separated by wide and rounded carinae with variable vertical development and inclination with respect to the corallum surface (Figures 4d, 6c). Where carinae separate, single calices or short series become distinct. In these cases, short carinae can resemble proximal cushions, the typical features forming in the agariciid genus *Leptoseris* when the inner or proximal side of an inclined corallite is raised into a cushion-like structure (Dinesen 1980).

*Columella*: Well-developed, sitting deep in the fossa (Figure 6d) made by one or more processes derived from the inner end of the radial elements.

**Figure 7.**
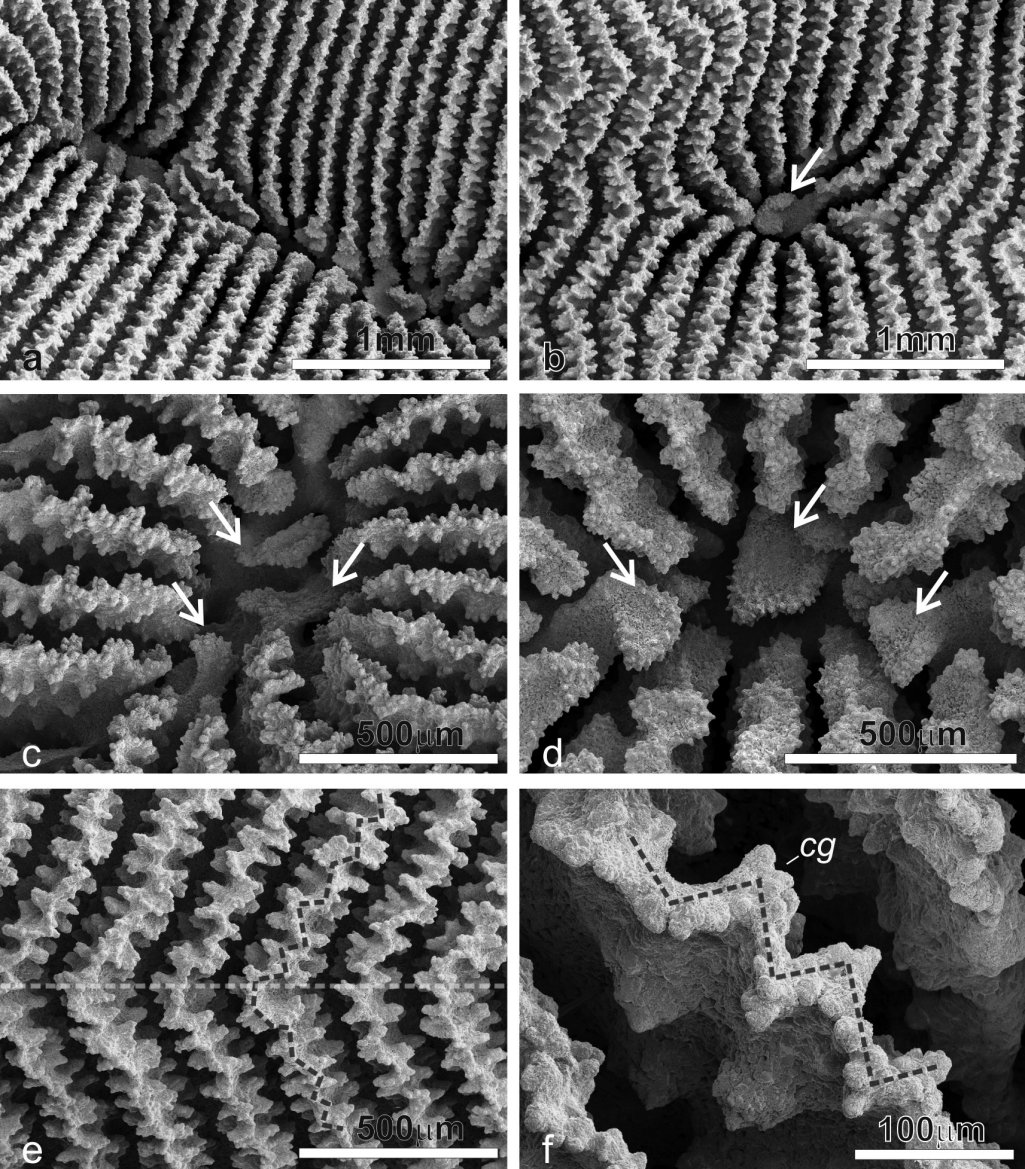
SEM images of *Pachyseris inattesa* sp. n. showing micro-morphological details. **a** Adjacent calices in series in specimen KAUST SA492 **b** Single calice in the same specimen as in **a**, the white arrow points at the single columellar process, which extends from the inner end of the radial elements reaching the fossa **c** Another calice in the same specimen (Figure 7a) with multiple columella processes (white arrows) extending from the inner end of the radial elements **d** A calice of specimen KAUST SA1305, the white arrows point at the finely ornamented columellar processes extending into the fossa from the inner end of the radial elements **e** Parallel and equal radial elements across a carina (top of the carina indicated by the dashed transparent white line) presenting the typical zigzag pattern of the margin (dashed grey line) **f** Detail of radial elements as in Figure 7e, showing the upper margin zigzag pattern (dashed grey line) and face ornamentation consisting of clumped granules (**cg**).

*Radial elements*: Radial elements are continuous across the carinae, regularly spaced and equal. Lateral faces bear regularly distributed, parallel lines of clumped granules (Figure 7e). The upper margin of the radial elements is minutely beaded and typically attains a zigzag pattern with ornamentations at the angles (Figure 7f).

*Color*: The *in vivo* color was light brown with the top of the collines and the margins of the colony paler.

##### Other material

**(MV Dream-Master, KAUST Biodiversity cruises, Saudi Arabia):** KAUST SA004, Al Lith (KBEF), 20°07.690'N, 40°12.513'E, 3 March 2013, coll. F. Benzoni; KAUST SA426, Qita al Kirsh, 22°25.597'N, 38°59.769'E, 18 March 2013, coll. F. Benzoni; KAUST SA429, Qita al Kirsh, 22°25.597'N, 38°59.769'E, 18 March 2013, coll. F. Benzoni; KAUST SA678, Al Wajh (KBEA), 25°23.515'N, 36°41.035'E, 25 September 2013, coll. F. Benzoni; KAUST SA860, Shibb al Hab (KBEA), 27°49.003'N, 35°06.397'E, 28 September 2013, coll. F. Benzoni; KAUST SA887, Jazirat Burcan (KBEA), 27°54.356'N, 35°03.555'E, 28 September 2013, coll. F. Benzoni; KAUST SA1284, Fsar, 22°13.779'N, 39°01.730'E, 20 October 2013, coll. F. Benzoni; KAUST SA1293, Fsar, 22°13.779'N, 39°01.730'E, 20 October 2013, coll. F. Benzoni and J. Bouwmeester; KAUST SA1300, Fsar, 22°13.779'N, 39°01.730'E, 20 October 2013, coll. F. Benzoni and J. Bouwmeester; KAUST SA1301, Fsar, 22°13.779'N, 39°01.730'E, 20 October 2013, coll. F. Benzoni and J. Bouwmeester; KAUST SA1305, Fsar, 22°13.779'N, 39°01.730'E, 20 October 2013, coll. F. Benzoni and J. Bouwmeester.

##### Variation of skeletal structures.

Average colony size is around 15 cm in diameter (Figure 4). The largest colony observed in the field was 25 cm across (Figure 11b). Corallum generally encrusting at the centre with foliose margins, thicker in colonies grown in well-lit environments and thinner in those from deeper and lower light conditions. Calices always distinct and arranged in series in most specimens although the length of the series can be very variable within and between specimens (Figures 4–6) and single calices can be also observed (Figure 6g). Carinae are always rounded, however they show much variation in height and width (Figure 4). Examples of the two ends of the wide variation range of the development of carinae in this species are provided in Figure 5. Columella always present, sitting low in the fossa, made of one or multiple spatula-shaped processes extending from the inner end of the radial elements (Figures 6a–d). No dissepiments were observed between the inner ends of the radial elements and the processes forming the columella in calices in series or alone (Figures 7a–b). Although radial elements are generally equal (Figures 6f–g, 7a–b) they can be unequal in some specimens (Figure 6e). Their faces’ ornamentation consists of parallel lines of clumped granules (Figures 7, 10a, c, e). Clumps of granules can fuse laterally to form short ledge-like features (Figure 10e), however these never develop into menianae *sensu stricto.* The upper margin of the radial elements is minutely beaded (Figures 7f, 10e) and typically attains a zigzag pattern with clumps of granules at the angles (Figure 7f).

##### Field characteristics and coloration.

In well-lit conditions and when growing on a horizontal substrate, this species tends to have a wrinkled appearance due to well-developed carinae. In colonies growing on inclined substrate and shaded conditions the carinae are less developed and the corallum surface can attain a smooth or slightly undulating surface. The coloration ranges from a grayish beige (Figures 4b, c, h, 5a–b) to brown with some areas having a greener tinge (Figures 4a, d–g, 5c–d).

##### Ecology.

*Pachyseris inattesa* sp. n. was recorded from different reef habitats between 10 and 35m depth. It grows on exposed reef slopes as well as in underneath overhangs and small caves.

##### Occurrence.

**Figure 8.**
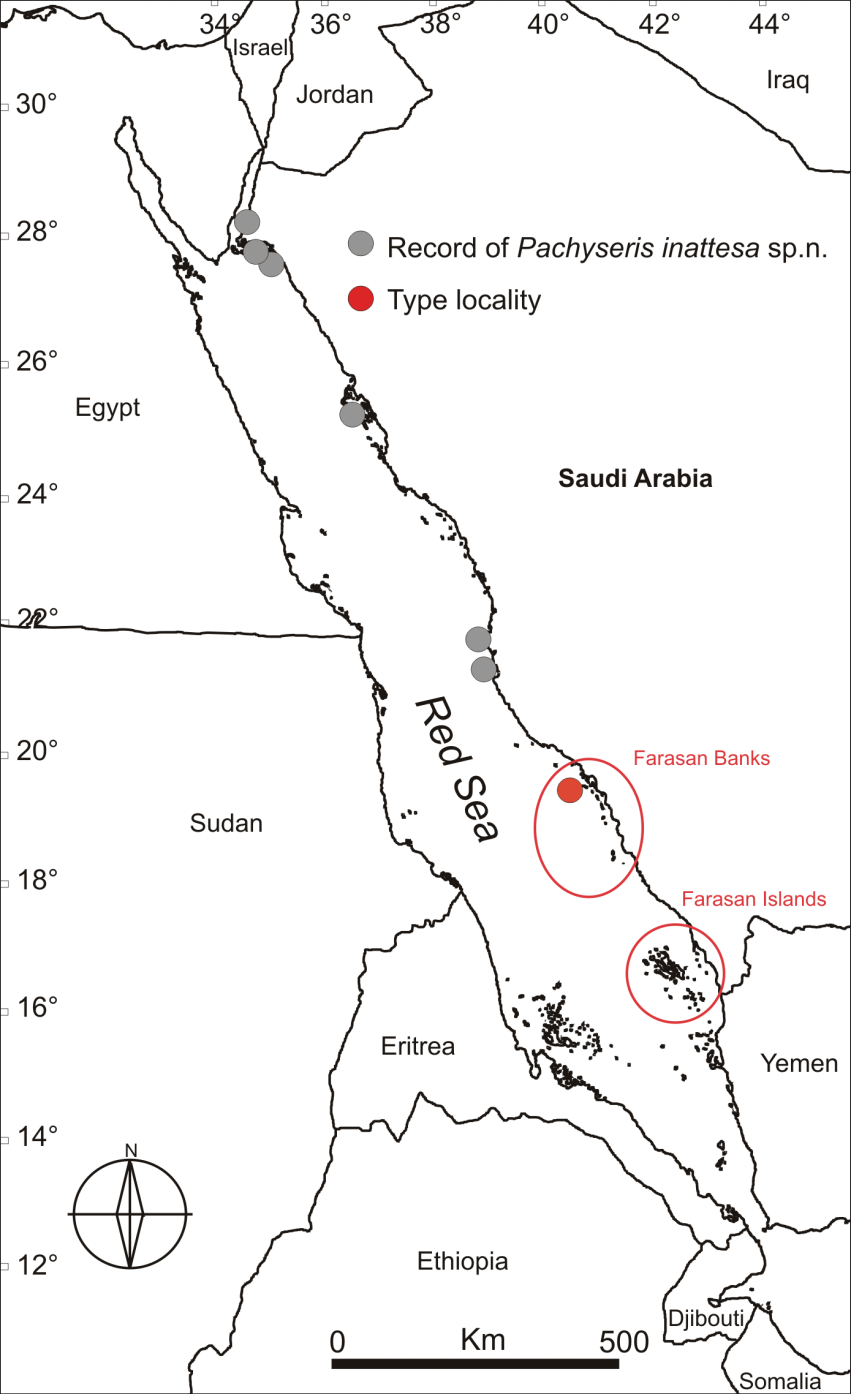
Map of the Red Sea showing the sampling sites where *Pachyseris inattesa* sp. n. was collected, including the type locality. Circled regions indicate approximate distribution of the Farasan Banks (a complex reef network spanning about ~250km of the Saudi Arabian coast) and the Farasan Islands (a system of > 80 islands spanning about 150km of the southernmost Saudi Arabian coast adjacent to the Yemeni border).

This species has been sampled along the Saudi Arabian coast in the northern and central Red Sea (Figure 8). It was not recorded in the Farasan Islands, nor further south in the Kamaran Islands, Yemen. To date, its distribution appears to be limited to the Red Sea.

##### Affinities.

Among its congeners, this species bears most resemblance to *Pachyseris speciosa*. However, with respect to the macro-morphology of the corallum, the corallites, and in corallite arrangement, this species is similar to and has been previously misidentified as *Leptoseris foliosa*.

##### Etymology.

*Inattesa* means “unforeseen” in Italian and stems from the initial bewilderment of the authors once they first examined the skeleton of the new species under a microscope.

## Results

### Phylogenetic analyses

**Figure 9.**
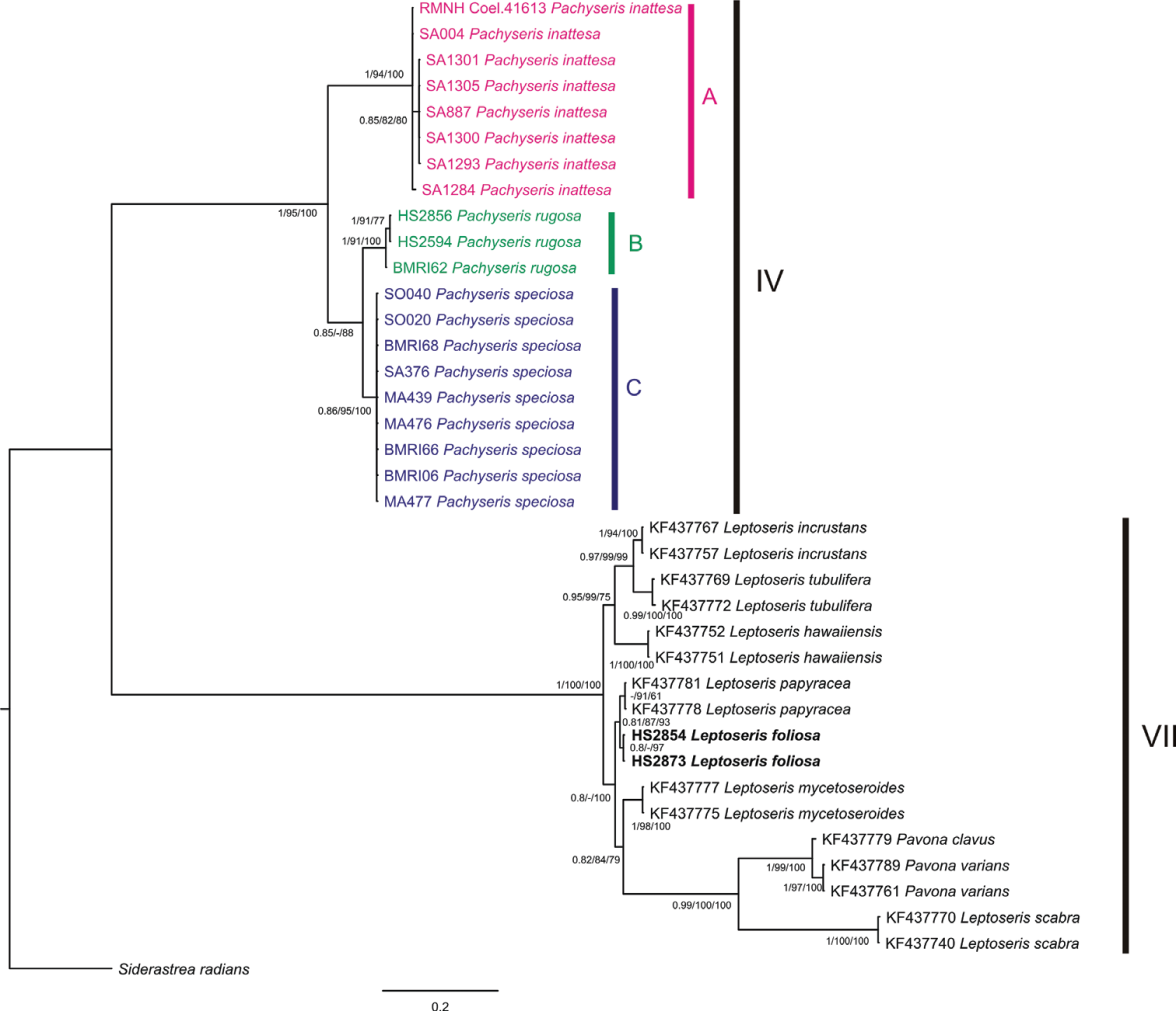
*Pachyseris* and Agariciidae phylogenetic reconstruction inferred from Bayesian inference analysis of mitochondrial intergenic spacer between COI and 16S-rRNA. Specimens identified as *Pachyseris inattesa* sp. n., *Pachyseris rugosa* and, *Pachyseris speciosa* are highlighted in pink, green and blue, respectively. Specimens of *Leptoseris foliosa* are indicated in bold. Uppercase letters A, B, and C delineate *Pachyseris* lineages. Clade numbers IV and VII are as reported by Fukami et al. (2008). Node values are Posterior Bayesian probabilities (>0.8), ML (>80%) bootstrap values, MP (>50%) bootstrap values. Posterior Bayesian probabilities below 0.8, ML bootstrap values below 80%, and MP bootstrap values below 50% are indicated by a dash (-). *Siderastrea radians* was selected as outgroup.

A total of 20 *Pachyseris* specimens belonging to three different species, namely *Pachyseris rugosa*, *Pachyseris speciosa*, and *Pachyseris inattesa* sp. n., and two *Leptoseris foliosa* sequences were successfully sequenced and used for phylogenetic reconstruction together with 15 agariciid sequences previously used by Luck et al. (2013). The final alignment consisted of 1,153 bp and included 83 variable sites, 76 of which were parsimony informative. A total of 124 mutations (considering only synonymous and non-synonymous substitutions) were found. Topologies resulting from BI, ML, and MP analyses were largely congruent with no contrasting signals (Figure 9) and showed high resolution at species level. Only the Bayesian phylogram with branch support indicated by Bayesian posterior probability (PP_BI_), ML bootstrapping support (BT_ML_), and MP bootstrapping support (BT_MP_) is shown in Figure 9.

The phylogenetic reconstruction resolved two main groups congruent with clade IV *sensu* Fukami et al. (2008) comprising *Pachyseris inattesa* sp. n. and the other two *Pachyseris* species, and clade VII *sensu* Fukami et al. (2008) composed by *Leptoseris foliosa* and the other representatives of the family Agariciidae (Figure 9). Within clade IV, *Pachyseris* specimens, including *Pachyseris inattesa* sp. n., were assigned to three well-supported main clades, namely A, B, and C. All the examined species belong to distinct lineages and their monophyly is highly supported (PP_BI_ = 1, BT_ML_ = 94, BT_MP_ = 100 for *Pachyseris inattesa* sp. n. in clade A, PP_BI_ = 1, BT_ML_=91, BT_MP_ = 100 for *Pachyseris rugosa* in clade B, PP_BI_ = 0.86, BT_ML_ = 95, BT_MP_ = 100 for *Pachyseris speciosa* in clade C) (Figure 9). The average intraspecific genetic distances for the examined *Pachyseris* species are very low, in particular 0.1 ± 0.1 % for *Pachyseris speciosa*, 0.7 ± 0.4 % for *Pachyseris rugosa*, and 0.1 ± 0.1 % for *Pachyseris inattesa* sp. n. The genetic distances between the three clades are higher, 10 ± 2.2 % between *Pachyseris speciosa* and *Pachyseris rugosa*, 16.5 ± 2.4 % between *Pachyseris speciosa* and *Pachyseris inattesa* sp. n., and 18.2 ± 2.8 % between *Pachyseris rugosa* and *Pachyseris inattesa* sp. n. The two sequences of *Leptoseris foliosa* were found in clade VII together with the other agariciid sequences from GenBank. In particular, *Leptoseris foliosa* is closely related to *Leptoseris papyracea* (Figure 9) and highly divergent from *Pachyseris inattesa* sp. n., a result supported by the genetic distance between the two species, 42.3 ± 3.1 %. Likewise, the genetic distance between *Pachyseris inattesa* sp. n. and *Leptoseris mycetoseroides* is also high, 42.3 ± 3.1 %.

## Discussion

### Affinities with *Pachyseris*

In a diagnosis of *Pachyseris*, Milne-Edwards and Haime (1851) remarked that the genus they described is characterized by “*Polypier semblable aux agaricies, si ce n'est que les polypiérites d'une même série sont complètement confondus entre eux*” [corallites similar to the agariciids, apart from the fact that the calices of the same series are completely confused between them]. *Pachyseris inattesa* sp. n. is, hence, quite untypical compared to the other species in the genus by having clearly recognizable calices, even when arranged in series. This is the most likely reason that the new species was previously identified as a *Leptoseris*. However, *Pachyseris inattesa* sp. n. is similar to the other two species of *Pachyseris* analyzed in this study on the basis of the arrangement of the carinae and the micro-morphology of the radial elements and of the columella. In particular, among its congeners, the new species is closer to *Pachyseris speciosa*. The two species have a similar growth form, although coralla of *Pachyseris inattesa* sp. n. do not form tiers. Furthermore, they have a similar structure of the columella, formed by minutely granulated processes extending from the inner ends of the radial elements into the fossa. However, the new species is devoid of the horizontal dissepiments uniting the radial elements with the columella. The most striking microstructural features shared by *Pachyseris inattesa* sp. n. and *Pachyseris speciosa* are the zigzag patterns of the upper margins of the radial elements, and their lateral faces’ ornamentation featuring clumps of granules, although the menianae and the vertically fused granules observed in *Pachyseris speciosa* do not form in *Pachyseris inattesa* sp. n.

### Dissimilarities with *Leptoseris*

The new species was previously collected in the Red Sea and identified as *Leptoseris tenuis* by Scheer and Pillai (1983: figs 7–11) and as *Leptoseris foliosa* by Sheppard and Sheppard (1991: fig. 85, fig 58). Moreover, in his description of *Leptoseris foliosa*, Veron (2000: p. 219, fig. 8) published an image of *Pachyseris inattesa* sp. n. from the Sinai Peninsula, Egypt. The author states that “Red Sea colonies are distinctive as they form thick figs which are usually flat”. Indeed, as mentioned above, colonies of *Pachyseris inattesa* sp. n. growing in well-lit conditions tend to be thick and to grow by following the underlying substrate (Figures 4c, 11b).

**Figure 10.**
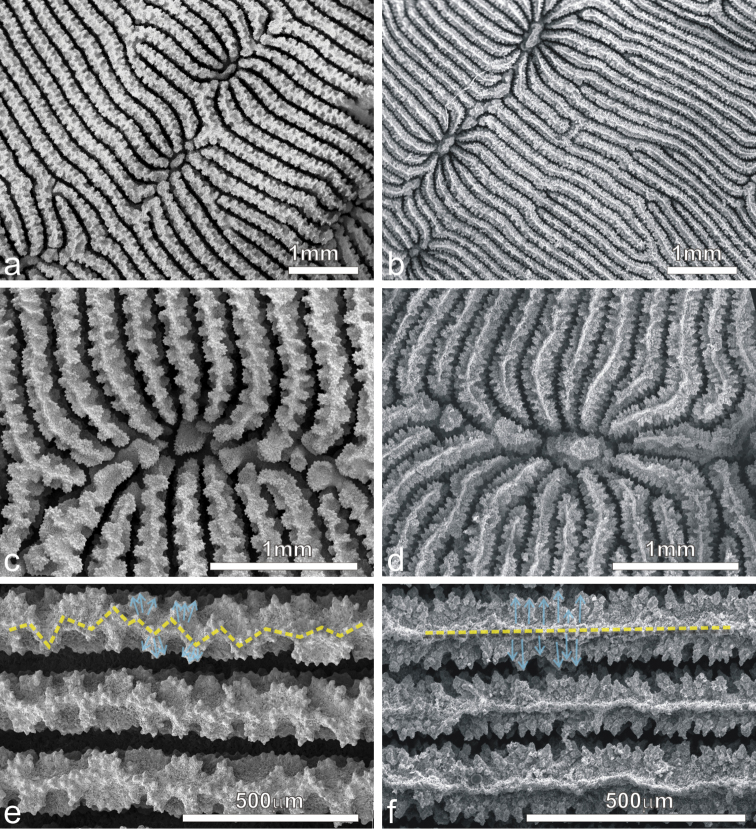
SEM images of *Pachyseris inattesa* sp. n. (a, c, e) and *Leptoseris foliosa* (b, d, f) **a** and **b** similar arrangement of adjacent calices in specimen KAUST SA1305 and IRD HS2854, respectively **c** and **d** calices the same specimens as in **a** and **b**, respectively **e** and **f** lateral ornamentation of the radial elements (light blue arrows) and pattern of their upper margin in specimens (yellow dashed lines) as in **a** and **b**, respectively.

The genus *Leptoseris* is characterized, like many other scleractinian taxa, by a great variability in the macro-morphology of the colonies and by a notable interspecific phenotypic variability that led to the identification of several nominal species and an enduring taxonomic confusion (Veron and Pichon 1980, Dinesen 1980, Luck et al. 2013). The two nominal species with which *Pachyseris inattesa* sp. n. has been confused, *Leptoseris tenuis* (Scheer and Pillai 1983) and *Leptoseris foliosa* (Sheppard and Sheppard 1991, Veron 2000), are a typical case of such a problematic taxonomic history. *Leptoseris foliosa* was described by Dinesen (1980) to include *Leptoseris tenuis* mainly because the holotype described by van der Horst (1921) was different from the actual specimen deposited as the holotype. Without engaging into an evaluation of the taxonomic action itself, it is clear that the same species was initially called *Leptoseris tenuis* and later *Leptoseris foliosa*. This species was recently examined in detail by Benzoni et al. (2012) who re-established *Craterastrea levis* Head, 1983, previously synonymized with *Leptoseris foliosa* as a result of molecular, micro-morphological, and microstructural analyses showing that the two nominal species were two distinct and valid taxonomic entities characterized by a striking convergence of traditional macro-morphological features. *Leptoseris foliosa* was included in the present paper in order to address its morphologic and molecular affinities with *Pachyseris inattesa* sp. n. Although the latter never forms upwards concave colonies as often observed in *Leptoseris foliosa* (Benzoni et al. 2012: Figure 10), the two species can form similarly shaped colonies (cf. Benzoni et al. 2012: Figure 8 with Figure 4b herein). However, despite a superficial similarity in terms of corallum shape, corallite arrangement in series (Figures 10a, b), and the formation of carinae separating series of calices, the micro-morphology of the radial elements and of the structure of the columella in *Pachyseris inattesa* sp. n. is substantially different from that of *Leptoseris foliosa* (Figures 10c–f). In *Pachyseris inattesa* sp. n. the columella is composed of one or more spatula-like processes extending from the margins of the radial elements into the fossa (Figure 10c), while in *Leptoseris foliosa* the columella is formed by one solid boss in all the corallites with the exception of the protocorallite (Benzoni et al. 2012) (Figure 10d). Moreover, while the upper margin of the radial elements in *Pachyseris inattesa* sp. n. is typically arranged in a zigzag fashion and the lateral ornamentation consists of clumps of radially arranged granules (Figure 10e), in *Leptoseris foliosa* the upper margin of the radial elements is straight and the granulations can be separated or elongated aggregations of parallel granules (Figure 10f), which can form or merge into menianae parallel to the growing septal margin (Benzoni et al. 2012: figs 28–32). Hence, similar to the case of *Leptoseris foliosa* and *Craterastrea levis*, and despite some macroscopic similarities we could find substantial micro-morphological differences substantiated by a significant genetic distance also between *Leptoseris foliosa* and *Pachyseris inattesa* sp. n. (Figure 9).

**Figure 11.**
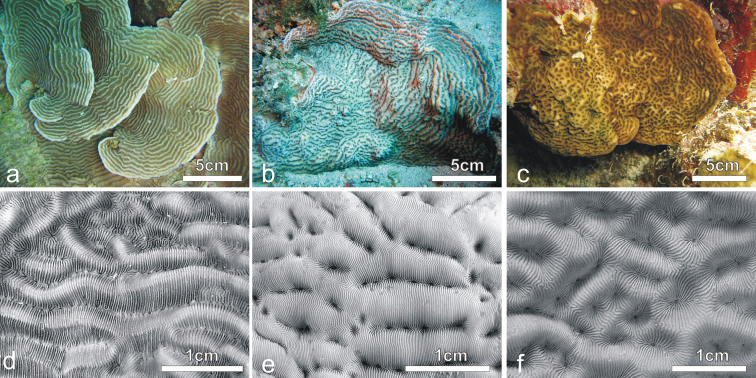
*In situ* and corallum images of **a** and **d** *Pachyseris speciosa* **b** and **e** *Pachyseris inattesa* sp. n., **c** and **f** *Leptoseris mycetoseroides* in the Red Sea.

*Leptoseris foliosa* was not encountered in the Saudi Arabian reefs in 2013, and since, as discussed above, previous records of this species in the Red Sea turned out to be *Pachyseris inattesa* sp. n., the presence of this species in the region is currently not confirmed. In the Red Sea *Pachyseris inattesa* sp. n. co-occurs with *Pachyseris speciosa* (Figures 11a–d) and with the common *Leptoseris mycetoseroides*. In the original description of this species, Wells (1954) noted some resemblance of this species to *Pachyseris*, such as the development of concentric carinae with *Pachyseris*-like rows of calices and the presence in some coralla of fig-like columellae (Veron and Pichon 1980). This species bears some superficial similarities with *Pachyseris inattesa* sp. n. in the colony morphology *in situ* (Figure 11c) but the typical ridges intersecting the carinae in *Leptoseris mycetoseroides* (Figure 11f) (Scheer and Pillai 1983) are never observed in the new species, which also has smaller calices and thinner and more numerous radial elements reaching the fossa (Figure 11). Our molecular results support these morphological gaps, revealing an important genetic distance between these two species (Figure 9).

### Molecular considerations

Although the mitochondrial genome of Scleractinia species is usually characterized by a slow evolution rate resulting in low levels of intraspecific variation (Shearer et al. 2002, Hellberg 2006, Huang et al. 2008), recent molecular studies have demonstrated that two mitochondrial intergenic non-coding regions, i.e., the putative control region located between ATP8 and COI and an open reading frame of yet unknown function located between ATP6 and NAD4 genes, exhibit high levels of sequence variation and can provide high resolution within the pocilloporid genera *Pocillopora* Lamarck, 1816, *Seriatopora* Lamarck, 1816, and *Stylophora* Schweigger, 1820 (Flot and Tillier 2007, Flot et al. 2008, 2011, Schmidt-Roach et al. 2012, Pinzón et al. 2013). Moreover, Luck et al. (2013) demonstrated that the mitochondrial spacer between COI and 16S-rRNA is powerful in resolving species boundaries in the agariciid genera *Pavona* and *Leptoseris*. In our study we present the first phylogenetic analysis of the genus *Pachyseris* based on the mitochondrial intergenic spacer between COI and 16S-rRNA. The region shows high variability and resolves species-level relationships within the genus.

The molecular phylogenetic reconstruction corroborates our micro-morphological results, confirming *Pachyseris inattesa* sp. n. as a monophyletic lineage belonging to *Pachyseris*, and not related to the family Agariciidae including the genus *Leptoseris*. This study confirms the utility of a combined morpho-molecular approach in resolving phylogenetic relationships among scleractinian species (Benzoni et al. 2007, 2011, 2014; Kitahara et al. 2012a, b; Huang et al. 2014; Arrigoni et al. 2014a, b).

### Biogeographic implications

As researchers increasingly combine traditional morphological with modern molecular taxonomic approaches on reference and museum collections (Rainbow 2009), previously overlooked taxa will continue to be discovered, or re-discovered (Arrigoni et al. 2014b) clarifying phylogenetic relationships and biogeography. This is particularly true in understudied regions such as the Red Sea, where corals and other organisms are currently being investigated for the first time through a morpho-molecular approach. For example, DiBattista and Randall (2013) have identified that a *Chromis* species (family Pomacentridae) once thought to be widespread through the Red Sea and Indian Ocean is actually two distinct species, one endemic to the Red Sea and one widespread throughout the Indian Ocean. Studies considering the evolutionary history of several fishes suggest that endemism in this region may be higher than currently thought (DiBattista et al. 2013).

The present study indicates that the same pattern may be true for a wider range of taxa including scleractinian corals, i.g., *Acropora hemprichii* (Ehrenberg, 1834), *Acropora variolosa* (Kluzinger, 1879), and *Cantharellus doederleini* (von Marenzeller, 1907) and provides some insight to more general biogeographic trends in the Red Sea (Hoeksema 1989, Wallace 1999). Specifically, *Pachyseris inattesa* sp. n. was not recorded in the Farasan Islands in the southern Red Sea (six sites), although the species was found in central and northern Red Sea sites (17), including the northern end of the Farasan Banks (nine sites) (Figure 8). Moreover, during the 2013 expeditions that led to the discovery of *Pachyseris inattesa* sp. n., remarkable differences in coral assemblages composition and discontinuities in species distribution were noted between the Farasan Banks and the Farasan Islands (Benzoni pers. comm). There is emerging evidence that the environmental shift between the Farasan Banks and the Farasan Islands (separated by ~110km) may represent a more general biogeographic barrier, with both a fish and a sponge species showing marked genetic structure correlated with environmental gradients (such as temperature, salinity, or productivity) between these habitats (Nanninga et al. 2014, Giles 2014).

Moreover, further investigations into the taxonomic status of marine species throughout the wider Red Sea, particularly with reference collections to facilitate morphological and molecular examinations (see Rocha et al. 2014), will help to delineate specific marine biogeographic province boundaries (as defined by Briggs 1974) as well as elucidate the evolutionary or ecological mechanisms involved. The role of the Red Sea in broader Indo-Pacific biogeographic patterns for scleractinian corals and other reef dwelling organisms remains intriguing (Bowen et al. 2013), potentially functioning as an exporter of biodiversity, i.e., a region in which some species have originated and then subsequently expanded their range beyond the Red Sea (e.g. DiBattista et al. 2013).

## Conclusion

*Pachyseris inattesa* sp. n. is described from the Saudi Arabian Red Sea based on a combination of morphological and molecular analyses. Although the macro-morphology of this species led to previous misidentifications with *Leptoseris tenuis* and *Leptoseris foliosa*, micro-morphological analyses revealed characters consistent with those of *Pachyseris* such as a concentric arrangement of the carinae, the structure of the columella (formed by extensions from the margins of the radial elements into the fossa), and a zigzag pattern of the upper radial elements. Molecular analyses of the mitochondrial non-coding spacer between COI and 16S-rRNA confirm that *Pachyseris inattesa* sp. n. is more closely related to other *Pachyseris* species than to the agariciid genera *Leptoseris* and *Pavona*.

## Supplementary Material

XML Treatment for Pachyseris

XML Treatment for Pachyseris rugosa

XML Treatment for Pachyseris speciosa

XML Treatment for Pachyseris inattesa
